# Gender Differences in Stress- and Burnout-Related Factors of University Professors

**DOI:** 10.1155/2020/6687358

**Published:** 2020-12-21

**Authors:** Laura Redondo-Flórez, José Francisco Tornero-Aguilera, Domingo Jesús Ramos-Campo, Vicente Javier Clemente-Suárez

**Affiliations:** ^1^Faculty of Sports Sciences, Universidad Europea de Madrid, Tajo Street, s/n, 28670 Madrid, Spain; ^2^Sport Science Faculty, Catholic University of Murcia, Murcia, Spain; ^3^Grupo de Investigación en Cultura, Educación y Sociedad, Universidad de la Costa, 080002 Barranquilla, Colombia

## Abstract

The aim of the present study was to analyse the gender differences in stress-related factors of university professors. A cross-sectional study was carried out, where gender differences in psychological, nutrition, physical activity, and oral health stress-related factors were analysed in 470 Spanish university professors (58.7% male and 41.3% female, 42.1 ± 9.2 years) through a compendium of questionnaires. The results showed how females presented significantly (*p* ≤ 0.05) higher scores than males in perceived stress (females: 22.15 ± 4.40 vs. males: 19.69 ± 3.61), emotional exhaustion (females: 20.86 ± 9.51 vs. males: 16.44 ± 9.12), and neuroticism (females: 5.53 ± 1.97 vs. males: 4.77 ± 1.96). These results may be related to higher probabilities to suffer the burnout syndrome, showing possible physical symptoms of this psychological disorder such as dry mouth and gastritis or heartburn. We concluded that female professors presented higher burnout perceived stress, emotional exhaustion, and neuroticism levels than males. Females also presented higher dry mouth, gastritis, and heartburn than males. Female professors showed healthier nutritional habits than males, presenting higher consumption of milk products and fruit per day, a higher number of meals, and less eating between hours and fried food consumption. Nevertheless, females consumed fewer water glasses and practised less weekly sport than male professors.

## 1. Introduction

Stress is a phylogenetic response triggered when a possible threat is perceived [1]. Stressors could be either external (from the context/environment, psychological, or social situations) or internal (illness, regeneration, recovery, and return to homeostasis processes). Stress may initiate the “fight or flight” response, implying a complex reaction of neurologic, endocrinologic, psychological, cognitive, and behavioural modifications, mostly regulated by the hypothalamus-pituitary-adrenal (HPA) gland axis and the autonomous nervous system [1, 2]. The hypothalamic response will lead to a release of corticotropin-releasing hormone (CRH) factor, which binds to CRH receptors on the anterior pituitary gland, releasing adrenocorticotropic hormone (ACTH); thus, receptors on the adrenal cortex stimulate the adrenal release of cortisol. In line with this, the autonomous nervous system is modulated, eliciting a sympathetic activation, leading to an increase of the heart rate, blood glucose, and blood pressure [3].

As an acute response, it is designed to maintain the physical integrity of the subject; however, the continuous stress exposition may derivate in psychopathologies as anxiety, depression, and posttraumatic stress disorder [4, 5]. In line with this, the educational context could be understood as stressful sustained stimuli, since burnout syndrome is a common psychopathology among this collective [6]. Burnout syndrome was defined as a typical labour stress form in which three dimensions were present: emotional exhaustion, depersonalization, and low professional accomplishment [7]. Thus, burnout syndrome and different personality traits which may predispose to this syndrome could be measured through different questionnaires [8]. Professors may feel emotionally exhausted, involving psychosomatic, behavioral, and emotional symptoms such as tiredness, frequent headaches, sleep problems, disorders in appetite and intake, irritability, impatience, depressive feelings, or inability to concentrate [9]. Therefore, burnout syndrome is considered as a negative work-related result when stress is not controlled [10]. In line with this, researchers linked labour stress with the appearance of physical and mental diseases, being related to approximately 50% of medical leaves [9]. Furthermore, university context has been found as a stressful ambient for both students [11–16] and professors [17] since participants showed a high sympathetic modulation related to the high demanding and eliciting context of education. Thus, due to the severity of burnout consequences in professors, diverse authors proposed the enforcement of emotional intelligence with the aim to facilitate the emotion processing and regulation as well as the achievement of successful stress management, which may help in burnout syndrome prevention [18–20]. There are several factors that could modulate the stress response. Physical activity has been postulated as one of these key factors, since regular exercise practice has been associated with significantly lower perceived stress levels [21–23]. This fact was explained by the increase of neurotransmitters, dopamine, serotonin, and endorphins, as well as its capability to modulate the autonomous nervous system [24–26]. In line with this, mind-body activities such as yoga and qigong seem to be also efficient for stress management [27]. Nutrition is another essential factor that may influence the stress response. Researchers highlighted how university students with a nonhealthy diet, based on high carbohydrates, saturated fats, and refined sugars, tend to have greater episodes of depression, anxiety, and perceived stress [28]. This is aggravated if there is a low consumption of greens, vegetables, and dairy and milk-related products [29] and fruits [30]. However, recent studies suggest that healthy eating is not correlated with stress reduction. Authors suggest that healthy eating might not be directly related to an instant relief in affect/stress, being possible that healthy eating impacts well-being with some delay, especially when physical consequences are its main driver [21]. Interestingly, greater levels of stress have been associated with worse oral hygiene and alterations in oral health behaviour which could be important in susceptible people since they might develop periodontal disease [31]. Also, researchers found associations between stress and the presence of plaque and gingivitis [32]. Finally, oral functional problems including chewing and speaking difficulties have been associated with mental health [33].

Actual studies have found discrepancies regarding gender differences in the stress response. In line with this, female professors showed higher levels of perceived stress than males [34], i.e., burnout syndrome is higher than in males [35, 36], and higher levels of emotional exhaustion [35]. Nevertheless, other authors found no significant differences between genders in their perceived stress [36]. Given the multifactorial basis of stress and the controversy in the scientific literature since no consistent findings conclude a clear influence of gender in professors' stress and burnout syndrome, we conducted the present multidisciplinary study with the aim to analyse gender differences in stress and burnout-related factors of university professors. The initial hypothesis was that female professors would present higher burnout levels and different psychological, nutritional, odontological, and physical activity behaviours than male professors.

## 2. Material and Methods

### 2.1. Participants

Four hundred and seventy university professors were interviewed in a period of six months, from January to June 2020, via online. We analysed the university teachers of Madrid province (Spain). They were advised via mail to participate in the present research, then they voluntarily decided to participate or not. The total number of university professors was 18000. The sample calculation with a 95% confidence level and 4.5 margin of error for this population was 462 participants. The specialization areas of professors that complete the research were Sciences, Health Sciences, Art and Humanities, Engineering and Architecture, and Social and Legal Sciences. All the procedures were conducted following the Helsinki Declaration (as revised in Brazil 2013) and approved by the University Ethics Committee (CIPI/18/074). All participants signed an informed consent before the start of the research.

### 2.2. Design and Procedure

A cross-sectional study was developed to reach the study aim. There was a call for teachers to voluntarily participate in filling the following questionaries.

#### 2.2.1. Teaching Activity Measures

*Teaching experience*. Was measured on a self-perception scale, indicating the years of professional experience in the university teaching service.

*Teaching satisfaction*. The item intends on a Likert scale from 0 to 10, where 0 is not all satisfied and 10 is completely satisfied, to indicate the professor's self-perception satisfaction in their job, considering their current and not past situation.

*Teaching stress*. As in the previous item, it is on a Likert scale from 0 to 10, where 0 is none and 10 is severe, which indicates the current stress self-perception.

#### 2.2.2. Psychological Measures

By the following questionnaires in their Spanish validated versions:

*Spanish version of the Maslach Burnout Inventory Test [* 37, 38*]*. This test assesses the presence of burnout syndrome. It is composed of 22 items that are answered on a six-point Likert scale, where 0 means never and 6 means daily. An example item is “I feel ‘burned' by the job.”

*Spanish version of the Perceived Stress Scale (PSS) [* 39, 40*]*. This scale assesses the level of perceived stress in a one-month period. It is composed of 14 items answered on a five-point Likert scale, meaning 0 means never and 4 means very often. An example item is “In the last month, how often have you felt upset about things that were out of your control?”

*Spanish version of the NEO Five-Factor Inventory (NEO-FFI) [* 41, 42*]*. This scale analyses five factors of personality: neuroticism, extraversion, openness, kindness, and responsibility. In the present research, we used a condensed version which included ten items answered by a five-point Likert scale, where 0 means strongly disagree and 4 means strongly agree. An example item is “I see myself as a person who is relaxed and tends to cope with stress.”

*Spanish version of the Acceptance and Action Questionnaire (AAQ-II) [* 43, 44*]*. This test analyses the psychological inflexibility or experiential avoidance through 7 items answered by a seven-point Likert scale, where 0 means never true and 7 means always true. An example item is “I worry about not being able to control my worries and feelings.” High scores suggest probable current clinical distress.

*Spanish version of the UCLA Loneliness Scale [* 45, 46*]*. This scale assesses the measurement of loneliness. In the present study, we used a condensed version composed of three items answered by a three-point Likert scale, where 1 means never and 3 means frequently. An example item is “How often do you feel left out?”

#### 2.2.3. Physical Activity Measures

This variable was analysed with a questionnaire used in line with previous researches which evaluated the psychophysiological stress response in high psychological demanding context [47, 48] which includes the following items: “How many hours are you physically active, in movement, per day?”; “Do you practise any endurance-based exercise (swimming, cycling, running…)? If yes, how long do you train per week?”; “Do you practise any sport team related exercise (football, basketball, volleyball…)? If yes, how long do you train per week?”; and “Do you practise any strength-based sport (weights, CrossFit, calisthenics ...). If yes, how long do you train per week?”. Questions should be answered by choosing the option offered which fits the best with the physical activity developed by the subject. According to the first question, “How many hours are you active, in movement, per day?”, answers ranged from “less than 30 minutes” to “more than three hours.” In the case of the rest of the questions, the answers range from “I do not practise this kind of training” to “more than 12 hours”.

#### 2.2.4. Nutrition Habit Measures

By an adapted previously used questionnaire [47, 49, 50] consisting of 20 questions, the first five were related to eating habits with dichotomous answers “yes or no.” The rest of the questions were related to the consumption frequency of different food groups, including meat, fish, vegetables, legumes, fast food, snacks, fried foods, soft drinks, water, or sweets, in which answers ranged from 1 to “more than 5.” Questions relating to daily hours of sleep and the number of days suffering illness per year are also included in this section.

#### 2.2.5. Oral Health Measures

By a previously used questionnaire [47, 49] consisting of six items related to oral health, the first four of them were answered by “yes, sometimes, or no.” The rest of the questions are “How many times a day do you brush your teeth?”, in which the answers ranged from “none” to “more than four,” and “Do you smoke?”, which ranged from “no” to “more than five cigarettes per day”.

### 2.3. Statistical Analysis

Statistical analyses were analysed using the Statistical Package for the Social Sciences (SPSS) version 24.0 (SPSS Inc., Chicago, Ill., USA). Descriptive statistics (mean and standard deviation) were calculated for each variable. Kolmogorov-Smirnov tests were performed to analyse the normality and homogeneity of each variable. A MANOVA with gender as a fixed factor was conducted to explore differences between university professors in the variables analysed. The level of significance was set at *p* < 0.05.

## 3. Results

Four hundred and seventy Spanish professors were analysed, 58.7% male and 41.3% female, mean age 42.1 ± 9.2 years. Data are presented as mean ± standard deviation. Males showed significantly greater height (*p* = 0.0001, *F* = 173.896), weight (*p* = 0.0001, *F* = 159.229), and body mass index (*p* = 0.0001, *F* = 33.896) while lower values of teaching stress (*p* = 0.0001, *F* = 25.45) than females. No significant differences were found between genders among teaching experience and teaching satisfaction scores (Table 1).

Males presented significantly higher values in personal fulfillment (*p* = 0.015, *F* = 5.971) than females. However, they showed lower values in emotional exhaustion (*p* = 0.0001, *F* = 12.708), perceived stress (*p* = 0.0001, *F* = 20.563), neuroticism (*p* = 0.004, *F* = 8.411), and conscientiousness (*p* = 0.002, *F* = 5.486) than females. No significant differences were found between genders among depersonalization, psychological inflexibility, loneliness, extraversion, agreeableness, and openness to experience values.

Regarding oral health items, females showed significantly higher values in daily toothbrushing (*p* = 0.044, *F* = 4.094), dry mouth (*p* = 0.015, *F* = 5.998), and gastritis or heartburn (*p* = 0.005, *F* = 3.885) than males. No significant differences were found between genders among gums bleeding, halitosis, and smoking habit scores (Table 2).

Males showed significantly higher values in the weekly team sport exercises (*p* = 0.019, *F* = 5.574) and the consumption of water glasses per day (*p* = 0.002, *F* = 10.013) and fried food per week (*p* = 0.002, *F* = 10.046) than females. Nevertheless, females showed significantly higher consumption of milk products (*p* = 0.013, *F* = 6.313) and fruit per day (*p* = 0.006, *F* = 7.845) than males. No significant differences were found between genders among daily movement, weekly endurance sport, weekly resistance training, illness days per year, hours of sleep, self-perception of eating healthy food, eating between hours, following a diet, reading of food labels, eating slowly, number of meals per day, and weekly consumption of sweets, meat, fish, legume, fast food, soft drink, and alcoholic drink scores (Table 3).

## 4. Discussion

The aim of the present study was to analyse the gender differences in stress- and burnout-related factors of university professors. The initial hypothesis was fulfilled since female professors showed higher burnout levels, as well as different psychological, nutrition, odontological, and physical activity behaviours than male professors.

In the present study, females presented higher levels of teaching stress than males. These results are consequent with previous literature since it was found how females suffered greater levels of burnout syndrome than males in their working environment [7, 51]. Regarding the educational context, previous authors found higher levels of perceived stress in female than male Spanish college professors [34]. However, in the present study, perceived stress values were greater compared to the research of Aparsi et al. (2019), probably explained due to the greater psychological demands imposed by the university and higher education context. In addition, regarding personality traits which may influence perceived stress and were considered one of the three dimensions of burnout syndrome, significantly greater values of emotional exhaustion were found in females, consequent with previous studies in Portuguese high school professors [36]. In line with this, a recent meta-analysis suggests that the gender gap regarding emotional exhaustion is greater in females. This trend seems to be significantly higher in US female employees when compared to Europeans; thus, a certain concern should be elicited by US organizations, policy-making institutions, and governing bodies alike [52].

Teaching stress could also be explained by the gap difference in other personality traits such as emotional exhaustion, neuroticism, and conscientiousness levels as well as in perceived stress, which were higher in females than in males, in addition to another personality trait such as personal fulfillment levels which were lower in females than in males. A psycho-emotional profile is highlighted as a common predictor of burnout [36]. These psychological constructs are related to maladaptive behaviours and psychopathologies such as anxiety or depression [53, 54], neuroticism being the aetiology and main characteristic of burnout [55]. Interestingly, despite women presenting greater teaching stress than males, no differences were found in teaching satisfaction. Authors suggest that usually, women tend to be happier than males at work; however, this trend is only reported in low-skilled jobs. In more complex settings and which require higher education, the differences in satisfaction between men and women tend to disappear [56], consequent with the present university professors' data. With these results regarding the psychological profile, we pointed out the influence of different personality traits in perceived stress and burnout syndrome.

Regarding the oral health stress factors, females showed significantly higher values in daily tooth brushing, dry mouth, and gastritis or heartburn than males. This high frequency of tooth brushing is consequent with previous researches [57] and may be related to the greater values showed by females regarding neuroticism and conscientiousness levels. Nevertheless, no significant associations were found between toothbrushing and psychological factors by other researches [58]. We found controversy relating to this issue. Regarding other stress-related factors associated with oral health, higher values of heartburn shown in the present research by females were also consistent with the previous literature of a study conducted in 60 subjects with current heartburn who suffered severe sustained life stress [59]. These greater values of stress have also been associated with gastritis [60], which is consequent with the gastritis shown by the present female participants. Indeed, previous authors claimed that sustained levels of stress are a risk factor of acute or chronic gastritis [61]. Interestingly, this association has been only found in females but not in males, in different animal models, with the result explained via the neuroendocrine pathway, suggesting that gastrointestinal inflammation has a gender-related impact on psychological behaviour, providing evidence for the existence of gastrointestinal-to-brain signalling [62]. According to these results, we also could consider the gastritis shown by female participants as a consequence of a sustained stress situation. Finally, dry mouth (xerostomia) has also been linked with greater stress perception, anxiety somatization, and depression [63–66], consequent with the psycho-emotional profile and stress perception of the present female sample. Thus, a relation between stress and oral health was found, wherein women tend to suffer more than males despite their high tooth brushing frequency, a fact in line with the previous bibliography in other collectives [32, 67].

In line with this, there was found how alterations on the oral microbiome have been related to gut dysbiosis, thus inducing changes in the composition of the gut microbiota, leading to changes in the production of proinflammatory cytokine and neurotransmitters that modulate the anxiogenic response [68, 69]. Also, previous researchers found how the larger perception of stress strongly influences the composition of the gut microbiota, leading to gastroesophageal reflux disease, peptic ulcers, functional gastrointestinal disorders, inflammatory bowel disease, and food allergy, by modulating motility, permeability, and visceral sensibility [70–72]. We can find how this vicious cycle is eminently caused by the perception and somatization of stress.

Regarding physical activity topics such as stress-related factors, in the present research, males presented significantly higher values of weekly team sport practise, which is consequent with previous studies regarding gender differences. Males tend to prefer collective and sport team activities than females [73]. However, goal orientations and attributional style have been somewhat inconsistent among the literature. Thus, it could be attributed to potentially confounding variables such as the level of participation or type of sport. Authors suggest that the psychometric characteristics of the subject play an important role, since individual sports are likely linked to higher ego orientation than team sports, with more internal, stable, global, and less externally controllable attributions for positive events, and more internal attributions for negative events than team sport athletes [74].

Regarding nutritional items such as stress-related factors, we found significant differences in hydration habits since males presented greater consumption of water glasses per day than females. However, females showed a significantly higher intake of milk products per day. In line with this, it has been highlighted how dairy product intake could perform as a powerful HPA axis modulator (pre- and probiotics products), as well as a gut modulator, this being linked to psychological disorders such as anxiety and depression [75–77]. Thus, it was also found how pre- and probiotics present in fruits and milk products modified gut microbiota composition due to their capability to enhance the growth of beneficial bacteria in the gut [78–80]. Significant differences were found in the present research relating to fruit consumption, showing female professors' higher intake than male professors. In line with this, our female participants could benefit more from this protective effect of pre- and probiotics in stress modulation than their male colleagues since their milk product and fruit consumption is significantly higher.

Female professors also showed higher meals per day and significantly lower consumption of fried food and eating between hours than males. According to different nutritional guidelines which pointed out fruit and vegetable intake recommendations (5 and 2 per day, respectively), the recommendation of avoiding the fried cooking process promoting boiling or steaming techniques [81], and the recommendation of making 5 meals per day and avoiding eating between hours [82], we found how females presented healthier nutritional habits than males. However, males showed higher practise of weekly team sport than females, a fact that could balance out their worse dietary habits, the results which were in consonance with previous research where a third of males mostly practised team sports [83].

## 5. Limitation of the Study and Future Research Lines

The main limitation of this study was the lack of biological measurement such as the gut microbial population and stress hormones (cortisol, adrenaline, alpha-amylase, etc.), a fact that may improve the complete understanding of stress-related effects on this population. Future studies may address these issues. Additionally, another limitation of this research is typical of cross-sectional studies, which is the inability of present causality, being only able to speak of association. Other drawings will be encouraged in the future. A further limitation of the study is the inability to extrapolate results to another country or other courses as we consider several differences could appear in the different cultural environment or teaching areas, both related to distinct stress context. Likewise, we consider the fact that stress was measured by the participants' self-perception and not by a validated scale which could be considered as an important limitation. Nevertheless, we used validated versions for psychological measures, and this was very positive. Finally, an additional one is the fact that we did not consider double workload, at work and at home, since nowadays, the female population still has to deal with some difficulties in the balance of family and work, and it may affect the perceived stress and burnout syndrome.

## 6. Practical Application

The multifactorial analysis of stress-related factors could be a useful tool to measure stress in university professors to explain and prevent the psychological consequences of burnout syndrome. Then, the use of questionnaires generates an easy source of information collecting data in a short period of time. The knowledge of these stress-related factors could be used by education institutions to implement multidisciplinary interventions to decrease stress and burnout in professors.

## 7. Conclusion

Female professors presented higher burnout perceived stress, emotional exhaustion, and neuroticism levels than males. Females also presented higher dry mouth, gastritis, and heartburn than males. Female professors showed healthier nutritional habits than males, presenting higher consumption of milk products and fruit per day, a higher number of meals, and less eating between hours and fried food consumption. Nevertheless, females consumed less water glasses and practised less weekly sport than male professors.

## Figures and Tables

**Table 1:** Body composition and teaching activity variables of male and female professors.

Variable	Male	Female	*F*	*p*
Age (years)	43.61 ± 9.36	41.03 ± 8.96	4.556	0.034
Height (cm)	176.76 ± 6.17	165.07 ± 7.02	173.896	0.0001
Weight (kg)	78.93 ± 10.35	62.18 ± 9.77	159.229	0.0001
Body mass index (kg/m^2^)	25.26 ± 3.11	22.80 ± 3.25	33.896	0.0001
Teaching experience (years)	12.07 ± 8.85	10.49 ± 8.20	1.978	0.161
Teaching satisfaction	8.37 ± 1.36	8.20 ± 1.34	0.88	0.349
Teaching stress	5.72 ± 2.25	7.16 ± 2.09	25.45	0.0001

**Table 2:** Psychology and oral health variables analysed in male and female professors.

Variables	Male	Female	*F*	*p*
Emotional exhaustion	16.44 ± 9.12	20.86 ± 9.51	12.708	0.0001
Depersonalization	4.77 ± 4.51	3.83 ± 3.70	3.051	0.082
Personal fulfillment	37.96 ± 6.28	36.07 ± 5.53	5.971	0.015
Perceived stress	19.69 ± 3.61	22.15 ± 4.40	20.563	0.0001
Psychological inflexibility	15.35 ± 8.31	16.13 ± 8.98	0.465	0.496
Loneliness	3.81 ± 1.26	3.97 ± 1.43	0.742	0.390
Extraversion	4.85 ± 1.74	4.93 ± 1.85	0.108	0.742
Agreeableness	7.39 ± 1.66	7.23 ± 1.43	0.619	0.432
Conscientiousness	8.20 ± 1.60	8.65 ± 1.30	5.486	0.002
Neuroticism	4.77 ± 1.96	5.53 ± 1.97	8.411	0.004
Openness to experience	8.13 ± 1.59	7.90 ± 1.77	1.024	0.313
Daily toothbrushing	2.58 ± 0.85	2.81 ± 0.82	4.094	0.044
Gums bleeding	0.38 ± 0.60	0.41 ± 0.61	0.153	0.696
Halitosis	0.25 ± 0.48	0.27 ± 0.46	0.079	0.779
Smoker	0.18 ± 0.65	0.25 ± 0.73	0.536	0.465
Dry mouth	0.35 ± 0.57	0.56 ± 0.71	5.998	0.015
Gastritis or heartburn	0.27 ± 0.51	0.44 ± 0.69	3.885	0.005

**Table 3:** Physical activity and nutrition variables measured in male and female professors.

Variable	Male	Female	*F*	*p* (sig)
Daily movement (h)	1.37 ± 1.10	1.52 ± 1.33	0.834	0.362
Weekly endurance sport (h)	1.23 ± 1.34	0.98 ± 1.39	1.912	0.168
Weekly team sport (h)	0.38 ± 0.84	0.15 ± 0.59	5.574	0.019
Weekly resistance training (h)	0.78 ± 1.16	0.65 ± 1.11	0.678	0.411
Illness days per year	5.93 ± 20.12	7.52 ± 19.69	0.366	0.546
Hours of sleep	6.64 ± 0.90	6.71 ± 0.98	0.289	0.592
Healthy food	1.18 ± 0.39	1.22 ± 0.41	0.524	0.470
Eat between hours	1.57 ± 0.49	1.44 ± 0.49	3.764	0.05
Follow a diet	1.84 ± 0.36	1.78 ± 0.40	1.149	0.285
Read food labels	1.40 ± 0.49	1.39 ± 0.48	0.027	0.869
Slowly eating	1.24 ± 0.43	1.30 ± 0.46	0.91	0.341
Meals per day	3.65 ± 0.88	3.88 ± 0.86	3.752	0.05
Water glasses per day	3.15 ± 3.10	2.10 ± 1.95	10.013	0.002
Milk products per day	1.85 ± 1.28	2.30 ± 1.39	6.313	0.013
Sweets per week	1.11 ± 0.80	1.09 ± 1.04	0.023	0.879
Meat per week	3.00 ± 1.42	3.05 ± 1.48	0.090	0.764
Fish per week	1.98 ± 0.98	2.26 ± 1.42	2.78	0.097
Legume per week	2.31 ± 1.27	2.13 ± 1.37	1.057	0.305
Fast food per week	0.88 ± 0.95	0.84 ± 0.98	0.128	0.721
Soft drink per week	1.37 ± 1.86	1.33 ± 1.99	0.022	0.883
Fried food per week	1.67 ± 1.26	1.17 ± 1.11	10.046	0.002
Daily fruit	2.44 ± 1.58	3.09 ± 1.83	7.845	0.006
Alcoholic drink per week	1.18 ± 1.1	1.02 ± 0.25	2.158	0.143

## Data Availability

All the data are in the manuscript.
